# Macrophage Heterogeneity in Respiratory Diseases

**DOI:** 10.1155/2013/769214

**Published:** 2013-02-27

**Authors:** Carian E. Boorsma, Christina Draijer, Barbro N. Melgert

**Affiliations:** ^1^Department of Pharmacokinetics, Toxicology and Targeting, Groningen Research Institute for Pharmacy, University of Groningen, Antonius Deusinglaan 1, 9713 AV Groningen, The Netherlands; ^2^GRIAC Research Institute, University Medical Center Groningen, University of Groningen, Hanzeplein 1, 9713 GZ Groningen, The Netherlands

## Abstract

Macrophages are among the most abundant cells in the respiratory tract, and they can have strikingly different phenotypes within this environment. Our knowledge of the different phenotypes and their functions in the lung is sketchy at best, but they appear to be linked to the protection of gas exchange against microbial threats and excessive tissue responses. Phenotypical changes of macrophages within the lung are found in many respiratory diseases including asthma, chronic obstructive pulmonary disease (COPD), and pulmonary fibrosis. This paper will give an overview of what macrophage phenotypes have been described, what their known functions are, what is known about their presence in the different obstructive and restrictive respiratory diseases (asthma, COPD, pulmonary fibrosis), and how they are thought to contribute to the etiology and resolution of these diseases.

## 1. Introduction

Most tissue macrophages are derived from hematopoietic stem cells and their local expansion within tissues can be due to local proliferation of existing macrophages or due to infiltration of blood-derived monocytes, depending on the circumstances. Traditionally characterized as the first line of defense against foreign invaders, research in the past decade has shown that their role extends to developmental processes and maintenance of tissue homeostasis in many ways [1, 2]. To fulfill these many different roles in tissue, macrophages can adopt a myriad of phenotypes based on signals they receive from their environment. From *in vitro* studies a nomenclature was proposed similar to the Th1/Th2 dichotomy, with M1 macrophages being known as classically activated macrophages induced by interferon gamma (IFN*γ*) and tumor necrosis factor alpha (TNF*α*) and M2 being known as alternatively activated macrophages induced by interleukin (IL)-4 and IL-13 [3, 4]. The M2 concept already had to expand to M2a, M2b, and M2c to encompass the many different phenotypes labeled alternatively activated, but these *in vitro* concepts have been hard to match to *in situ* tissue macrophages. This is in part caused by a lack of specific markers for the different phenotypes within tissue and by the observation that *in situ* macrophage phenotypes appear as a continuum rather than discrete entities [5, 6].

Macrophages are among the most abundant cells in the respiratory tract and can be broadly divided into two populations depending on their localization: alveolar macrophages (AMs) that line the surface of alveoli and interstitial macrophages (IMs) that reside in the space between alveolar epithelium and vascular endothelium [7]. It has been suggested that AM do not originate directly from blood monocytes, but instead are derived from IMs which therefore serve as an intermediate between blood monocytes and AMs. Compared with AMs, IMs are less efficient in phagocytosing but are better at stimulating T-cell proliferation *in vitro* [8]. In addition, IMs as opposed to AMs, were also found to produce high levels of IL-10 and thereby inhibit DC migration [9]. Although IMs and AMs have distinct functions, they both are among the first to encounter allergens and other threats to the lung homeostasis [8, 10, 11]. They are both capable of quickly dealing with those without perturbing normal gas exchange because they can adopt the most effective phenotypes based on signals from surrounding tissue. These phenotypical changes are also linked to many respiratory diseases. In both obstructive (asthma, COPD) and restrictive respiratory diseases (pulmonary fibrosis) changes in the number and phenotype of lung macrophages have been found. In this paper we will first briefly discuss the *in vitro* generated phenotypes and then compare this with their role in the pathogenesis of obstructive and restrictive respiratory diseases.

## 2. M1, M2, and Beyond

### 2.1. M1 Macrophages

**Figure 1:**
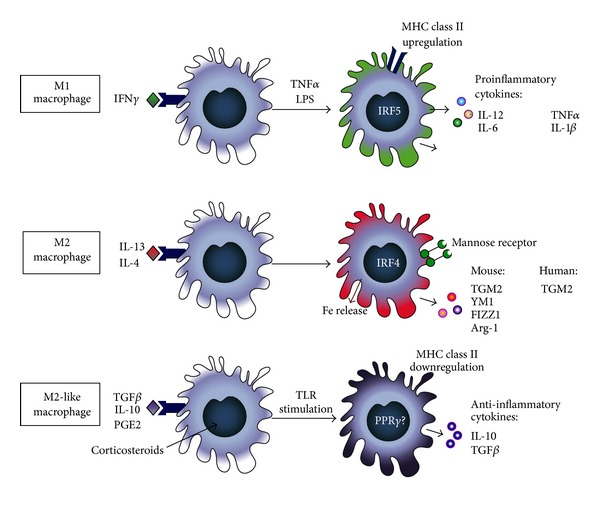
Schematic representation of the three macrophage phenotypes and their characteristics. IFN*γ*: interferon gamma; TNF*α*: tumor necrosis factor alpha; LPS: lipopolysaccharide; MHC class II: major histocompatibility complex class II; IL: interleukin; NO: nitric oxide; IRF5: interferon regulatory factor 5; Fe: iron; TGM2: transglutaminase 2; YM1: chitinase-3-like protein-3; FIZZ1/Relm*α*: resistin-like molecule-*α*; Arg-1: arginase-1; TGF*β*: transforming growth factor beta; TLR: Toll-like receptor; PGE2: prostaglandin E2; PPAR*γ*: peroxisome proliferator-activated receptor gamma.

Classically activated or M1 macrophages develop after being exposed to IFN*γ* and TNF*α* or lipopolysaccharide (LPS, which induces TNF*α* production) under the influence of the transcription factor interferon-regulatory factor 5 (IRF5) [12]. They are essential in host defense against intracellular pathogens by generating reactive oxygen species (ROS) and nitric oxide (NO) through upregulated expression of inducible nitric oxide synthetase (iNOS) and amplifying Th1 immune responses by producing proinflammatory cytokines like IL-12, IL-1*β*, and TNF*α* (see also Figure 1) [13]. In addition, they show enhanced phagocytosis of microorganisms, antigen-presentation capabilities, and enhanced production and secretion of matrix metalloproteinases (MMPs) such as MMP7 and MMP9 [14–17]. The secretion of MMPs enables macrophage migration during inflammatory responses, but excessive or unregulated production results in tissue damage [5, 17]. 

### 2.2. Alternative Activation

Alternatively activated or M2 macrophages were named to indicate that their activation status was distinctly different from the classically activated macrophages. First discovered to be induced by IL-4 and IL-13 [18, 19], this phenotype was soon found to have more siblings, closely resembling each other but distinctly different in function [5, 20]. A variety of different names have been suggested, but for the purpose of this paper we will adopt the names suggested by Mosser and Edwards [5] and Sica and Mantovani [20]. They have suggested alternatively activated or M2 macrophages for the phenotype induced by IL-4/IL-13 and regulatory macrophages or M2-like cells for the phenotype characterized by high IL-10 production that are induced by a variety of stimuli (see also Figure 1).

### 2.3. M2 Macrophages

M2 macrophages, induced by IL-4/IL-13 under the influence of the transcription factor IRF4 [21], have a role in protection against helminths and are considered wound-healing macrophages because of their association with physiological and pathological tissue remodeling [5, 22]. They are characterized by upregulated expression of mannose receptors and transglutaminase 2 in man and mice [19, 23] and by upregulated expression of arginase-1, chitinase-3-like protein-3 (Chi3l3, also known as Ym1), and resistin-like molecule-*α* (Relm*α*, also known as FIZZ1) in mice only (see also Figure 1) [22, 24, 25]. They have poor antigen presenting capabilities and exhibit increased release of iron and clearance of apoptotic cells and extracellular matrix components (efferocytosis) [26–28].

### 2.4. M2-Like Macrophages

M2-like macrophages also upregulate mannose receptors and in addition produce high levels of IL-10 (see also Figure 1). They are induced by a number of stimuli that need to be combined with a second signal, which is Toll-like receptor (TLR) stimulation. The initial signals include glucocorticosteroids, prostaglandin E2 (PGE2), antibody immune complexes, transforming growth factor beta (TGF*β*), and IL-10 itself [5]. They may also be the macrophages that produce TGF*β* in addition to IL-10, but this has not been rigorously shown due to the overlap in markers between M2 and M2-like macrophages [29–32].

Transcriptional control of this phenotype is unclear but may involve peroxisome proliferator-activated receptor gamma (PPAR*γ*) and the cAMP-responsive element-binding protein (CREB)-CCAAT/enhancer-binding protein-*β* (C/EBP*β*)-axis [33]. As a result of their high IL-10 production, M2-like macrophages have strong anti-inflammatory activity. This can be beneficial during later stages of immune responses to limit inflammation but may also permit tumor progression when associated with tumors [5, 13]. To date it has been difficult to distinguish genuine M2 macrophages from M2-like macrophages because they share many markers, most notably the mannose receptor. Only IL-10 production would be a reliable marker but is used seldomly to identify M2-like macrophages [5]. The exact differences in tissue distribution and function of these two phenotypes are therefore difficult to establish from the studies published to date. Whenever possible, we will indicate what is known of M2 and M2-like macrophages in the context of respiratory diseases.

## 3. Macrophages and Asthma

### 3.1. Asthma

Over the last few decades the prevalence of asthma has rapidly increased, and currently more than half a million people suffer from asthma in The Netherlands (Annual Report 2011 Dutch Lung Fund). More women are affected by this underdiagnosed and undertreated airway disease than men. Asthma is a heterogeneous disorder of the airways, which are chronically inflamed and contract easily in response to nonspecific stimuli. This so-called airway hyperreactivity is accompanied by increased mucus secretion and airway wall remodeling, which leads to symptoms such as wheezing, coughing, and chest tightness [34].

Several distinct forms of asthma have been recognized and can roughly be divided into atopic and the less-studied nonatopic asthma. The majority of asthma patients are atopic, which is a predisposition to mount an immunoglobulin type E (IgE) response. This type is characterized by infiltration of eosinophils in the lungs. In nonatopic asthma there is no evidence of allergen-specific IgE, and this type is characterized by the infiltration of neutrophils in the lungs. A small portion of asthma patients suffer from severe asthma, which includes both atopic and nonatopic characteristics. Severe asthma is defined as being unable to control asthma symptoms despite taking high-dose corticosteroids, also referred to as corticosteroid-resistant asthma [35]. 

### 3.2. Pathogenesis of Asthma

Asthma is traditionally considered a T-helper-2- (Th2-) cell driven inflammatory disorder. Activation of a Th2-response is characterized by the release of the cytokines IL-4, IL-5, IL-9, and IL-13. These Th2-cytokines are responsible for the recruitment of effector cells resulting in eosinophil infiltrates, IgE production, and histamine release among other typical asthma symptoms. The innate immune system is increasingly being recognized as an additional important disease mechanism in asthma [36]. Cells of the innate immune system actively orchestrate adaptive immune responses in asthma [37]. Besides dendritic cells (DC) in the lung taking up allergens and pathogens and presenting those to the adaptive immune system, other cells important for innate immune responses in the lung are macrophages. Their role, however, in asthma has been greatly underestimated, and therefore their contribution to asthma is mostly unexplored [38].

### 3.3. Macrophage Phenotypes and Asthma

In asthma it appears that effective phenotype switching is impaired and macrophages can actually contribute to the pathogenesis of this disease. The next part will focus on the roles of each known phenotype in the pathogenesis of asthma.

### 3.4. M1 Macrophages in Asthma

Although the inflammatory process in asthma is dominated by a Th2 inflammation, increasing evidence supports the parallel development and involvement of both M1 and M2 macrophages in this disease. We have recently shown that during the development of house-dust-mite-induced asthma numbers of M1 macrophages are high in a short model as compared to control mice and decrease with longer exposure [39]. Levels of M1 inducers (IFN*γ* and LPS or TNF*α*) were found to be significantly higher in asthmatics, especially in those with severe forms of the disease [40–42]. Elevated serum IFN*γ* correlates with the severity of airway inflammation in atopic asthma, and this cytokine has been linked to mechanisms that induce airway hyperreactivity [43, 44]. In agreement with the findings in human asthma, it was shown that both IFN*γ* and LPS contribute to airway inflammation and airway hyperreactivity in a mouse model of asthma [45, 46]. TNF*α* is implicated in many aspects of asthma pathology, including development of airway hyperreactivity and attraction of eosinophils and neutrophils [47, 48]. 

In both atopic and nonatopic asthmatics, the amount of LPS in house dust has been related to the severity of airway inflammation [49, 50]. Inhalation of pure LPS by asthmatics is associated with bronchoconstriction and a change in airway hyperreactivity [51, 52]. Administration of high doses of LPS into the lungs of allergic mice promotes airway hyperreactivity, neutrophilic inflammation, and expression of M1 cytokine IL-12. In addition, exposure of asthmatic mice to both IFN*γ* and LPS induced higher numbers of macrophages in the lungs [40].

M1 macrophages polarize under the influence of the transcription factor IRF5. It was shown that a common *IRF5* gain-of-function haplotype is associated with asthma and the severity of asthmatic symptoms. These associations were more pronounced in nonatopic asthmatics, and it was suggested that IRF5 may only have a profound impact on the pathogenesis and severity of nonatopic asthma and not on atopic asthma [53]. An explanation could be that M1 macrophages are responsible for the recruitment of neutrophils, which are the major effector cells in nonatopic asthma. Neutrophils are also dominant in more severe phenotypes of asthma, and the most commonly used therapy for asthma, corticosteroids, is not effective against neutrophilic inflammation [54]. This is in accordance with recent findings that corticosteroid-resistant asthmatics have increased expression of M1 markers on macrophages in bronchoalveolar lavage fluid (BALF) compared to corticosteroid-sensitive asthmatics, suggesting that M1 macrophages also play a key role in the development of severe corticosteroid-resistant asthma [55]. 

A few studies have shown that M1 macrophages act preventively in the *onset* of allergic airway inflammation in mice [56, 57] and suppressed DC maturation [58]. This is consistent with the findings of a study that investigated the role of the M1 cytokine IL-12 during the development allergic airway inflammation in mice. They showed that neutralization of IL-12 during the sensitization phase aggravated development of allergic airway inflammation but neutralization of IL-12 during challenges abolished the development of allergic airway inflammation. These data demonstrate a dual role of IL-12: it acts preventive during Th2 sensitization, but it contributes to allergic airway disease during allergen challenges. The effects of IL-12 neutralization were not shown in IFN*γ* knockout mice, suggesting that IFN*γ* plays an essential role in the IL-12-induced effect [59].

Thus, both the presence of M1 skewing factors (IFN*γ*, TNF*α*, or LPS) and the proinflammatory mediators released by M1 macrophages can contribute to asthma. The data imply that M1 macrophages may be beneficial to prevent allergic sensitization, but in already established disease they promote the development of M2 macrophages and induce corticosteroid resistance. Besides a role in severe asthma, markers of M1 macrophages have also been implicated in nonatopic asthma. 

### 3.5. M2 Macrophages in Asthma

The cytokines IL-4 and IL-13 are abundantly present in the lungs of asthmatics, and it may therefore not come as a surprise that markers expressed by M2 macrophages have been associated with asthma. Elevated levels of chitinase family members have been found in the serum and lungs of patients with asthma, suggesting the presence of increased M2 macrophage numbers [60, 61]. Indeed, we showed that asthmatics have higher percentages of macrophages expressing mannose receptor and transglutaminase 2 in bronchial biopsies than in healthy subjects [23, 62]. In addition Kim et al. showed that severe asthmatics had higher numbers of IL-13-positive M2 macrophages in BALF as compared to healthy controls [63]. Both chitinase levels and the percentage of mannose receptor-positive macrophages also correlated with asthma severity [60, 62]. Higher numbers of M2 macrophages were also found in children undergoing severe exacerbations of asthma [64]. In addition, we have recently shown in several models of house-dust-mite-induced asthma that the number of M2 macrophages positively correlated with the severity of airway inflammation [39]. These clinical and animal model findings demonstrate a correlation between asthma severity and the number of M2 macrophages, but it is unclear whether M2 macrophages actively contribute to the induction and exacerbation of the disease or are just bystanders in allergic airway inflammation responding to the high IL-4 and IL-13 levels.

Credit to the role of M2 macrophages in the exacerbation of the disease was given by adoptive transfer studies. The transfer of *in vitro* differentiated M2 macrophages into the airways of male asthmatic mice aggravated airway inflammation [65]. Another study using IL-4R*α*-positive M2 macrophages showed that intraperitoneal injection of these macrophages was sufficient to increase the allergic inflammatory response in the lung [66]. In a different model of fungus-induced asthma, Moreira et al. showed that transfer of M2 macrophages into the lungs of mice enhanced both inflammation and collagen deposition [67] as compared to asthmatic mice not treated with macrophages. Since M2 macrophages and their products have been reported in asthma patients, M2 macrophages may be a target to reduce asthma symptoms. Indeed, the study by Moreira et al. in mice with fungus-induced asthma also showed that treatment with an inhibitor of M2 macrophage generation resulted in lower airway hyperreactivity, mucus cell proliferation, collagen deposition, and M2 numbers as compared to control mice [67]. In support of these results, inhibition of M2-expressed transglutaminase 2 reduced ovalbumin-induced airway hyperreactivity, ovalbumin-specific IgE levels, and infiltration of inflammatory cells in lung tissue [68]. These studies substantiated previous circumstantial evidence concerning a role for M2 macrophages in the pathogenesis of asthma [69, 70]. 

Unfortunately, the previous studies did not conclusively prove that M2 macrophages play a causative role in the development of allergic airway inflammation. In contrast to what has just been described, Nieuwenhuizen et al. recently demonstrated that M2 macrophages are not necessary for allergic airway disease and may only be a consequence of the elevated Th2 response. They studied the contribution of M2 macrophages to acute, chronic, and house-dust-mite-induced allergic airway inflammation by using mice with abrogated IL-4R*α* signaling on macrophages. It was demonstrated that airway hyperreactivity, Th2 responses, mucus hypersecretion, number of eosinophils, and collagen deposition were not significantly affected by decreased development of M2 macrophages. However, the expression of M2 markers was still higher in mice with macrophage-restricted IL-4 receptor-*α* (IL-4R*α*) deficiency as compared to healthy mice. The presence of these small numbers of M2 macrophages may still have been able to reinforce the Th2 response [71]. 

To sum up, M2 markers are correlated with severity of allergic airway disease in humans and mice, suggesting that M2 macrophages contribute to the disease. Indeed, elimination of M2 macrophages in established disease by pharmacological interventions remarkably decreased the degree of airway inflammation. However, new data suggest that M2 macrophages are not essential for the development of allergic airway inflammation but only play a bystander role as a consequence of the Th2 response.

### 3.6. M2-Like Macrophages in Asthma

Reports on the role of M2-like macrophages in asthma are few. These macrophages could play an important role in the resolution of asthma because of their production of IL-10. Interestingly, a lower level of IL-10 production was found in lung macrophages from asthmatics compared to healthy persons [72]. Moreover, macrophages from severe asthmatics produce high levels of IL-6 and IL-8, but IL-10 was undetectable in these cells compared to macrophages from patients with moderate asthma [73].

Studies in mouse models of allergic airway inflammation have investigated the role of IL-10 intensively and found it to be an important mediator in the resolution of airway inflammation [74], but only few studied the production of IL-10 by macrophages. We have just shown that the number of IL-10-positive cells is lower in lungs of mice with house-dust-mite-induced asthma as compared to control mice [39], and recently it was also shown that lung interstitial macrophages produce high levels of IL-10 and prevent airway inflammation in mice [9]. Stimulation of macrophages by ovalbumin uptake and TLR ligands induced increased production of IL-10 by these macrophages, and this resulted in lower levels of IL-5 and ovalbumin-specific IgE and a lower number of eosinophils in a mouse model of asthma [75]. 

Although evidence for a role of M2-like macrophages in asthma is scarce, these findings suggest a protective effect since active IL-10 production by these cells is low in moderate asthma and absent in severe asthma. In a mouse model of asthma IL-10 was shown to act as an anti-inflammatory. Studies on the resolution of asthma may reveal whether an increased production of IL-10 by these macrophages is involved.

**Figure 2:**
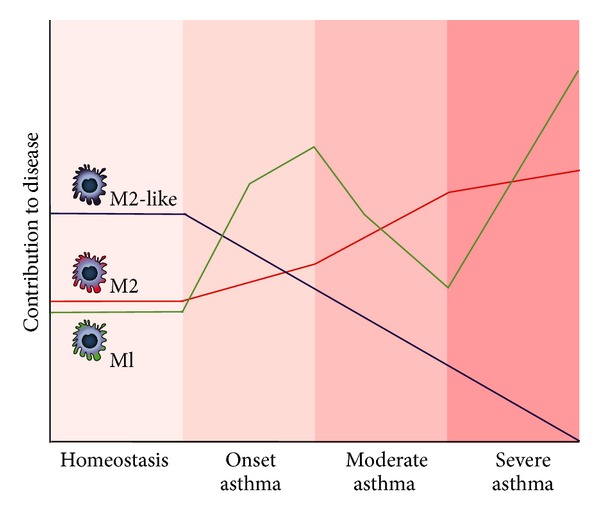
Schematic representation of the presence of M1, M2, and M2-like macrophages in lung tissue during homeostatic conditions, induction of asthma, and during moderate and severe asthma.

Combining the data available for the different subsets in asthma (see also Figure 2) suggests that M1 macrophages can prevent the induction of asthma but during established disease can cause severe corticosteroid-resistant asthma. M2 macrophages are associated with asthma and their presence correlates with more severe disease. However, it is still a matter of debate whether they genuinely contribute to asthma pathogenesis or are just innocent bystanders of the inflammation. M2-like macrophages seem to be beneficial to the resolution of asthma through production of IL-10 but are not present or not functional in asthma, and therefore allergic inflammation can progress.

## 4. Macrophages and Chronic Obstructive Pulmonary Disease (COPD)

### 4.1. COPD

COPD is one of the most common respiratory diseases and affects around 320,000 people in The Netherlands (Annual Report 2011 Dutch Lung Fund). It is projected to be the fourth leading cause of death worldwide by 2030 and places a huge economic burden on society [76]. COPD is caused by lung inflammation due to inhalation of noxious gasses and particles: in the Western World most commonly from cigarette smoking and in developing countries from indoor biomass cooking and heating [77]. The disease is characterized by airflow limitation that is not fully reversible, which is caused by a combination of obstructive bronchiolitis (also known as chronic bronchitis) and destruction of alveoli resulting in airspace enlargement (also known as emphysema) [78]. The relative contributions of chronic bronchitis and emphysema to the COPD phenotype can vary from person to person. 

### 4.2. Pathogenesis of COPD

Exposure to smoke and particles leads to an exaggerated chronic inflammation in lungs of people susceptible to the development of COPD. Excess mucus production and progressive narrowing of the respiratory bronchioles characterize chronic bronchitis. The mucosa, submucosa, and glandular tissue become infiltrated with inflammatory cells and the walls of the respiratory bronchioles become thickened because of edema and fibrosis [79]. Chronic mucus hypersecretion is induced by goblet cell hyperplasia and hypertrophy of submucosal glands [80], which further contributes to occlusion of small airways. This progressive narrowing leads to obliteration or even complete disappearance of respiratory bronchioles. Not much is known about the role of macrophages in this part of the disease, but pigmented macrophages were found to cluster around small airways and these were associated with peribronchiolar fibrosis [81]. 

The alveolar destruction that characterizes emphysema is the result of infiltration of inflammatory cells with a prominent role for macrophages. Both neutrophils and macrophages are being recruited to the lung because smoke/particle exposure injures epithelial cells that subsequently release cytokines and chemokines to recruit them [82, 83]. They have been postulated to be the main effector cells contributing to the excess tissue damage seen in emphysema because of their ability to produce proteolytic MMPs like neutrophil elastase and macrophage elastase (MMP12) [83, 84]. Increased numbers of macrophages and neutrophils have been found in airways and lung parenchyma of patients with COPD [85–87]. However, only the number of parenchymal alveolar macrophages was directly proportional to the severity of lung destruction in emphysematous lung tissue from COPD patients [88]. Animal studies confirmed the dominant role for macrophages, because deletion of neutrophils in smoke-exposed rats did not prevent cigarette smoke-induced emphysema, whereas deletion of macrophages did [89]. In addition, mice deficient in MMP12 (mainly produced by macrophages) were completely protected from cigarette-smoke-induced emphysema even though they could still produce neutrophil elastase [90]. Similarly, inhibiting MMP12 reduced smoke-induced airway inflammation in mice [91].

### 4.3. Macrophage Phenotypes and COPD

The role of the different macrophage phenotypes in COPD is the topic of quite a few studies recently and the subject of much debate as the results have been somewhat counterintuitive. Based on studies in mice and results from patient studies, M1 polarization is expected to play an important role in the pathogenesis of COPD. However, the results of other studies have questioned this view, and this is nicely illustrated by studies from Shaykhiev et al. and Hodge et al. [92, 93]. The first ones recently studied the transcriptome of alveolar macrophages from healthy smokers and nonsmokers and compared them to alveolar macrophages from COPD smokers [92]. Their results showed a mixed phenotype for alveolar macrophages after smoking with downregulation of M1 genes and partial upregulation of M2 genes, which was progressively worse in COPD. Hodge et al. showed a mixed phenotype in alveolar macrophages of smoking COPD patients with some M1 (MHC II expression) and M2 (efferocytosis) markers going down and some going up (proinflammatory cytokine production and DC-SIGN expression) [93]. In the next part we will touch upon this debate as we discuss the separate phenotypes in the pathogenesis of COPD.

### 4.4. M1 Macrophages in COPD

Several lines of evidence support not only a role for M1 macrophages but also a role for dysregulated M1 macrophages in the development of COPD. First of all, exposure to compounds in smoke appears to induce M1 polarization of macrophages. Smoking is the most important risk factor for COPD and cigarette smoke contains many thousands of compounds, including LPS that can activate macrophages in the lung [94]. Indeed, increased expression of iNOS in alveolar macrophages was found in COPD patients [95–97], indicating a polarization towards an M1 phenotype. Upregulation of iNOS increases ROS and NO production and can then cause oxidative stress. Oxidative stress has been shown to be an important contributor to the pathogenesis of COPD [98]. Smoking itself of course causes oxidative stress, and increased iNOS activity through M1 polarization can add to this stress [99–101].

Furthermore, many studies have shown that smoke exposure enhances the release of the M1 proinflammatory cytokines IL-1*β*, IL-6, IL-8, and TNF*α* [102–107]. M1-derived cytokines also play a role in the pathogenesis of COPD. IL-1*β*, IL-6, IL-8, and TNF*α* have all been found to be elevated in COPD [108–118] and in experimental settings have been found to contribute to the development of persistent airway inflammation, emphysema, and mucus production [102, 119–124]. TNF*α* was found to drive most of the emphysema development in mice after smoking because mice lacking receptors for TNF*α* only developed mild emphysema [124]. In addition, mice overexpressing TNF*α* in lung tissue develop chronic inflammation and emphysema [119, 125, 126]. However, in humans antibodies against TNF*α* seem to be ineffective in COPD, questioning the relevance of this cytokine for human COPD [127]. In addition to TNF*α*, M1 cytokine IL-1*β* was also found to play a role [102, 120]. Overexpression of IL-1*β* in lung caused lung inflammation, emphysema, mucus metaplasia, and airway fibrosis in mice [121]. Taken together these data suggest cytokines produced by M1 macrophages at least play a role in the pathogenesis of COPD.

Another important M1-related cytokine with a role in COPD is IFN*γ*. It is produced by CD8+ T cells that infiltrate the lungs in COPD [88, 128, 129] and can cause M1 polarization. Inducible overexpression of IFN*γ* in lungs of mice caused emphysema with alterations in the balance of MMPs and antiproteases [53]. However, in human alveolar macrophages from smokers reduced expression of IFN*γ* receptors and reduced IFN*γ* signaling were found, suggesting M1 polarization may be impaired after smoking. This of course is in line with the above-cited finding by Shaykhiev et al. that M1 genes are downregulated in alveolar macrophages of healthy smokers and smoking COPD patients as compared to nonsmokers [92].

M1 macrophages have also been found to produce MMP9, presumably to enable macrophage migration during inflammatory responses [5, 17]. MMP9 is associated with the breakdown of extracellular matrix in COPD as macrophages from patients with COPD have a significantly higher production of MMP9 as compared to control macrophages [130]. In addition, overexpression of human MMP9 in mouse macrophages induced emphysema and loss of alveolar elastin pointing at a role for M1 macrophages in COPD development [131]. 

Finally, an important property of M1 macrophages that appears to be dysregulated is phagocytosis of microorganisms. M1 macrophages are geared towards killing and disposal of microbial threats and phagocytosis of microorganisms is part of that function [14, 15]. COPD is often exacerbated by infections [132], and there is accumulating evidence that reduced macrophage phagocytosis in COPD may be responsible for the persistence of microorganisms in the lungs [133–135]. This dysfunction of phagocytosis is not restricted to microorganisms but also appears to be present for M2-related phagocytic functions such as efferocytosis and mannose receptor-mediated uptake [136, 137]. This overall inhibition of phagocytosis irrespective of macrophage phenotype was further confirmed by the later study of Hodge et al. that has already been mentioned before [93].

Taken together, the available data suggest that a dysregulated M1 response plays a role in COPD rather than an increased number of M1 macrophages. Some aspects of the M1 activation signature are upregulated in COPD (ROS generation, proinflammatory cytokines, production of MMP9), but some aspects are also downregulated (phagocytosis, IFN*γ* responsiveness).

### 4.5. M2 Macrophages in COPD

Overexpression of prototypical M1-inducer IFN*γ* may be able to induce emphysema, but so does overexpression of prototypical M2 induced IL-13. Zheng et al. showed that mice overexpressing IL-13 in lung tissue caused lung pathology mirroring human COPD with macrophage- and lymphocyte-rich inflammation, emphysema, and mucus metaplasia [138]. Unfortunately, macrophages were not further characterized in this study, so it is not known if IL-13 overexpression also induced more alternative activation of macrophages. Further evidence for a role for M2 macrophages came from a study by Kim et al. who showed that viral infections could induce an IL-13-producing M2 phenotype through interactions with natural killer T cells leading to chronic airway inflammation [63]. They also showed higher numbers of IL-13-positive M2 macrophages in lung tissue of COPD patients. 

In mice, M2 macrophages produce large amounts of chitinases like Ym1 and Ym2 [139]. Whether their human counterparts are also induced by alternative activation is unclear, but another member of this family, stabilin-1 interacting chitinase-like protein (SI-CLP), has been found upregulated in M2 macrophages [140]. Whether or not pointing at alternative activation, many members of the chitinase family associate with COPD. Chitotriosidase levels, for instance, were increased in bronchoalveolar lavage of smokers with COPD and they also had more chitotriosidase-positive cells in bronchial biopsies and an elevated proportion of alveolar macrophages expressing chitotriosidase as compared to smokers without COPD or never smokers [141]. Furthermore, macrophage chitinase-1 was selectively increased in a subset of patients with severe COPD [142], and serum concentrations of YKL-40 were significantly higher in smokers with COPD as compared to nonsmokers or smokers without COPD and correlated negatively with lung function [143–145]. Interestingly, YKL-40 also stimulated the production of proinflammatory cytokines and MMP9 by macrophages from COPD patients, suggesting YKL-40 itself actually induces more of an M1 phenotype [145].

Data from studies investigating MMPs indicate a possible role for M2 macrophages. As mentioned above MMP12 plays an important role in mouse emphysema [90, 91], and MMP12 was found specifically induced in IL-4-stimulated M2 macrophages [146]. Furthermore, Woodruff et al. showed increased M2 polarization of alveolar macrophages in smokers using MMP12 as a marker for alternative activation [147], and many others showed that smoke induces MMP12 in macrophages [148–154]. Interestingly, MMP12 production by macrophages was also found to be necessary to terminate both neutrophil and macrophage influx at the end of an inflammatory response and may therefore be an instrument of M2 macrophages to dampen inflammation to be able to start remodeling of damaged tissue [155]. How that ties in with the potential proemphysematous role of M2 macrophages remains an open question.

Summarizing, there is some evidence for a role of M2 activation in COPD, and this evidence points at a role contributing to the development of COPD. The data by Hodge et al. suggest that, similar to dysfunctional M1 activation, M2 activation is also dysregulated with reduced efferocytosis but increased expression of M2 marker DC-SIGN [93]. 

### 4.6. M2-Like Macrophages in COPD

No attempts have been made to distinguish the roles of M2 and M2-like macrophages in COPD. Two studies reported IL-10 in the context of COPD. A study by Hackett et al. showed diminished IL-10 production in lung tissue of COPD patients after LPS stimulation as compared to lung tissue of patients with normal lung function [156]. Takanashi et al. demonstrated that the level of IL-10 and the number of IL-10-positive macrophages in sputum from COPD patients and healthy smokers was decreased as compared to healthy nonsmokers [157]. This would suggest that M2-like macrophages are impaired in smoking and COPD and therefore cannot suppress the ongoing inflammation induced by smoke.

**Figure 3:**
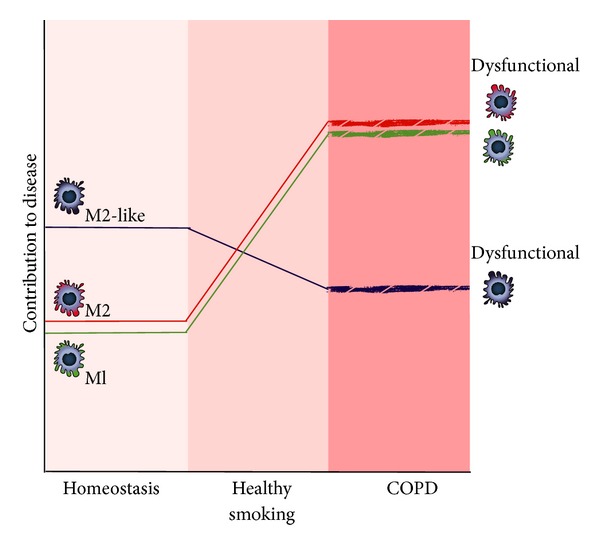
Schematic representation of the presence of M1, M2, and M2-like macrophages in lung tissue during homeostatic conditions, during healthy smoking, and in COPD. Please note the dysfunctional state of macrophages during COPD.

Combining the data available for M1, M2, and M2-like macrophages (see also Figure 3), it appears COPD is a disease of dysfunctional macrophages rather than a disease of one particular polarization state. Macrophages in COPD are promoting ongoing inflammation and tissue damage but are unable to effectively dampen inflammation because they have lost the ability to phagocytose microorganisms and apoptotic bodies and produce anti-inflammatory cytokines like IL-10.

## 5. Macrophages and Pulmonary Fibrosis

### 5.1. Pulmonary Fibrosis

Pulmonary fibrosis is a disease that encompasses a collection of restrictive pulmonary disorders characterized by progressive and irreversible destruction of lung architecture by excessive deposition of extracellular matrix (ECM) [158]. While ECM formation usually functions as an essential process of tissue healing after lung injury, continuous damage may result in abnormal wound repair and progress to fibrosis. Fibrosis of the interstitium ultimately leads to organ malfunction because of the disturbed architecture of the lung, causing impaired gas exchange and eventually death from respiratory failure [158]. In some cases, fibrotic lesions remain localized to a limited area of the lung because the initial trigger is removed, for example after tuberculosis or a fungal infection, while in others such as in sarcoidosis and idiopathic pulmonary fibrosis (IPF) the fibrotic process continues to progress throughout the lungs in a diffuse manner [159].

IPF is the most common and most dangerous of the fibrotic lung diseases. The chronic and slowly progressing character of the disease together with an unknown aetiology makes it a difficult disease to diagnose and treat. The incidence of IPF appears to be increasing and is currently estimated at 7–16 cases per 100,000 persons [160]. Patients diagnosed with IPF have a poor life expectancy with a median survival of 2–5 years [161]. Currently there are no effective therapies available for these patients, as no therapy has yet been proven to cure or even halt the progression of fibrosis [159]. 

### 5.2. Pathogenesis of Pulmonary Fibrosis

To describe the pathogenesis of pulmonary fibrosis and to be able to unravel the complex interactions of macrophages, tissue repair after injury can be divided into four different stages: the clotting phase for emergency tissue repair, then the inflammatory phase to fight the inciting agent, followed by formation of scar tissue in the fibrotic phase for more permanent repair, and eventually resolution of scar tissue and restoration of tissue homeostasis in the resolution phase. During fibrosis some or all of these stages are dysregulated as will be discussed below. 

Pulmonary fibrosis is thought to be the result of repetitive injury to the epithelial cell layer lining the alveoli. This damage initiates a blood coagulation cascade to prevent severe blood loss and to maintain some sort of homeostasis. This includes platelet accumulation and production of fibrin by epithelial cells, which is essential for fibrin-containing clot formation [162]. To restore the function of damaged tissue, plasminogen activator (PA) eventually breaks down this fibrin matrix again. In pulmonary fibrosis, changes in both the coagulation cascade itself and the resolution of the wound-healing clot can affect the disease. Impaired fibrin degradation for instance has been shown to worsen epithelial cell survival [163]. Impaired resolution of clots can be caused by either the absence of PA [164] or by increased production of PA inhibitors PAI-1 or PAI-2 [165]. 

Cell damage furthermore triggers an inflammatory reaction in lung tissue. It has been difficult to investigate the role of the former and this phase in fibrosis because patients usually present with end-stage disease. Nevertheless, the inflammatory response has been extensively studied in LPS-induced inflammation in humans (reviewed by Rossol et al. [166]). It was shown that epithelial cell damage induces the release of several cytokines and chemokines that triggers an influx of neutrophils, closely followed by monocytes to fight the inciting agent [167]. Epithelial cells also release growth factors like TGF*β*, TNF*α*, and epidermal growth factor alfa (EGF*α*) that stimulate tissue healing by activating fibroblasts, which are the main producers of collagen and other ECM proteins [168, 169].

Control of the inflammatory event, however, is essential for a proper wound healing process [169]. Dysregulation of the inflammatory phase with a prominent role for M1 macrophages has long been thought to be important to the process of fibrosis. The fact that anti-inflammatory drugs such as corticosteroids have no therapeutic effects in patients with pulmonary fibrosis has made this assumption unlikely [170]. Now the new prevailing hypothesis is that pulmonary fibrosis probably develops when the fibrotic phase and/or resolution phase become dysregulated [171]. 

To progress from the inflammatory phase to the next phase of tissue repair, inflammation needs to be dampened. The release of IL-10 and TGF*β* dampens inflammation and promotes ECM production by myofibroblasts [172]. Under the influence of TGF*β* and PDGF produced by damaged epithelial cells and platelets, fibroblasts differentiate into myofibroblasts, proliferate, and produce ECM proteins [173]. Furthermore, they start producing their own TGF*β* to maintain tissue healing [174]. In pulmonary fibrosis this phase is probably dysregulated as increased numbers of myofibroblasts and increased production of ECM are found in fibrotic lungs. Increased numbers of M2 macrophages are also associated with this phase, and these macrophages are therefore suggested to play an important role in the development of fibrosis [169].

Eventually repair of the epithelial cell barrier and removal of excess ECM are essential to recover normal lung function. To overcome the loss of alveolar epithelial type I cells (AEC I), alveolar epithelial type II cells (AEC II) become hyperplastic and provisionally restore the epithelial cell layer along with the ECM produced by myofibroblasts [168]. Normally these type II cells would revert back to AEC I and homeostasis is restored. However, when injury is repetitive this does not seem to occur; ECM is produced continuously and AEC II continue to proliferate without reverting back to AEC I. In a proper tissue healing response, the excess of ECM products is removed to gain full function of the lungs again. Macrophages are important cells in degrading and taking up ECM components. In order to do so they produce MMPs and their inhibitors (tissue inhibitors of metalloproteinases, TIMPs). A balance between the activities of MMPs and TIMPs is important to maintain tissue homeostasis [175]. Levels of both MMPs and TIMPs are elevated in patients and mouse models of pulmonary fibrosis [176], but their balance is clearly disrupted as the net result is an excess of ECM in those lungs.

### 5.3. Macrophages in Pulmonary Fibrosis

Macrophages play an important role in the pathogenesis of lung fibrosis, but their role is complex. They are involved in many of the dysregulated tissue healing responses in fibrosis, and they can also adopt many phenotypes. This complexes studies into their role in fibrosis tremendously. In the next part we will discuss what is known about the contribution of each macrophage phenotype to each stage of fibrosis.

### 5.4. M1 Macrophages in Pulmonary Fibrosis

We have found no studies reporting on the presence of M1 macrophages in pulmonary fibrosis except for one study by Nagai et al. showing that folate-receptor-beta- (FR*β*-) positive macrophages were higher in patients with IPF as compared to controls [177]. These macrophages have previously been shown to produce TNF*α* and oxygen radicals and are therefore very likely M1 macrophages [178].

Several lines of evidence suggest that M1 macrophages may play a role in both the inflammatory phase as well as the resolution phase of pulmonary fibrosis. As a reaction towards epithelial cell damage, monocytes are recruited to the site of inflammation and differentiate into M1 macrophages under the influence of proinflammatory cytokines. Once activated, M1 macrophages themselves produce TNF*α*, IL-1*β*, and oxygen radicals to kill and phagocytose microbes to fight an infection or remove an exogenous agent [179]. Many studies indicate that these proinflammatory cytokines and oxygen radicals are associated with fibrosis development [180–188]. In the study by Nagai et al. ablation of the FR*β*-expressing M1 macrophages during the inflammatory phase of bleomycin-induced fibrosis abrogated fibrosis development [177]. However, the importance of the contribution of inflammation to established fibrosis has been challenged because anti-inflammatory drugs such as corticosteroids have no therapeutic effects in patients with pulmonary fibrosis [170]. This view was confirmed by a study from Gibbons et al. They studied newly recruited inflammatory macrophages in a mouse model of bleomycin-induced lung fibrosis and showed that depletion of tissue-resident macrophages and/or circulating inflammatory monocytes during the inflammatory phase did not affect the onset or degree of fibrosis that developed after this inflammatory phase [189]. Another study pointed out that the M1 cytokine TNF*α* has beneficial effects on alveolar epithelial cell recovery and therefore also contributes to resolution [190].

In the resolution phase, macrophages are involved in the degradation of excess ECM and the uptake of matrix components [189, 191]. Depletion of macrophages during this recovery phase impaired the resolution of fibrosis by slowing down the degradation of ECM [189]. It is unclear what type of macrophages is responsible for degradation of ECM, but a case can be made for M1 macrophages as these have been shown to produce several MMPs including MMP7 and MMP9. Levels of MMP9 have been found to be increased in lungs of IPF patients and this may reflect a failing attempt of the lungs to remove excess ECM and may be caused by a simultaneous increase of the inhibitor TIMP-1 [192–194]. 

Macrophages are also important in the subsequent removal of ECM components through endocytosis-mediated mechanisms. Again it is unclear if this is restricted to one particular phenotype, but the receptors involved would suggest more of an M2 phenotype, and this will therefore be discussed in the next part on M2 macrophages. 

In summary, M1 macrophages are important in the inflammatory phase, but their presence does not appear to affect the subsequent fibrotic phase. During resolution of scar tissue, macrophages are indispensable for degradation of ECM. This may be related to an M1 phenotype, and it may therefore be beneficial to stimulate recruitment of M1 macrophages to reverse fibrosis. 

### 5.5. M2 Macrophages in Pulmonary Fibrosis

There is a great deal of evidence that Th2 responses are important in the development of fibrosis, and it appears that IL-13 is the predominant cytokine in the profibrotic responses [195–202]. Levels of IL-13 are higher in patients with pulmonary fibrosis as compared to controls, and macrophages isolated from these fibrotic lungs produce more IL-13 than macrophages from control lungs [203]. It therefore comes as no surprise that M2 macrophages are associated with pulmonary fibrosis, although we could not find publications directly showing numbers of M2 macrophages are increased in lung tissue of patients with pulmonary fibrosis. We did find one study showing higher numbers of M2 macrophages in BALF of IPF patients as compared to controls and two studies showing higher numbers of insulin-like growth factor-I (IGF-I)-positive and PDGF-positive interstitial macrophages in lung tissue of IPF patients as compared to controls [204–206]. Both these markers are important profibrotic mediators, and a recent study by Chen et al. showed that expression of IGF-I colocalized with arginase-1 and not with IL-10 expression in macrophages suggesting genuine M2 macrophages express IGF-I and not the M2-like subset [207]. This was a study in mice; it therefore remains to be investigated whether this is also true in humans.

Markers found on or produced by M2 macrophages have also been found to be increased in pulmonary fibrosis. Levels of galectin-3, a carbohydrate-binding lectin that is necessary for alternative activation [208], were higher in BALF of IPF patients as compared to control patients [209]. Furthermore, macrophages from IPF patients produced more of the human M2 marker CCL18 than control macrophages, and this correlated negatively with pulmonary function test parameters [210]. IPF patients were also found to have higher serum and pulmonary levels of chitinase-like protein YKL-40 as compared to controls, although it is still unclear whether chitinases are true markers of alternative activation in human macrophages [211]. This is also the case for arginase-1, which is a marker of M2 macrophages in mice but its specificity in humans is debated [22]. Nevertheless, lung tissue from IPF patients had higher expression of arginase-1 in macrophages than normal lung tissue [212]. Lastly, circulating monocytes from systemic sclerosis patients with pulmonary fibrosis showed enhanced profibrotic phenotype by increased expression of CD163, a marker of alternative activation in humans [213].

Experimental models of pulmonary fibrosis have revealed more about the role of M2 macrophages in fibrosis of the lung. Depletion of macrophages during the fibrotic phase of lung fibrosis reduced the deposition of ECM in this organ [189]. To confirm a role for M2 macrophages, levels of Ym1 and arginase-1 were measured before and after macrophage depletion. Both markers showed decreased expression in the lungs after removal of macrophages. The M1 marker iNOS did not show a reduction in expression, indicating that M2 macrophages are predominantly responsible for the development of fibrosis. Furthermore, M2 marker MMP12 was shown to be essential in the development of fibrosis induced by excessive activation of Fas [214] and in a model of IL-13 dependent fibrosis [215].

There is some evidence or how M2 macrophages would contributes to the fibrosis development. The aforementioned production of IGF-I and PDGF contribute to proliferation of fibroblasts and their transformation to ECM-producing myofibroblasts [173]. Furthermore, FIZZ1 (also known as resistin-like molecule alpha) was found to increase ECM production in fibroblasts [216], but a recent paper by Pesce et al. showed that FIZZ1 actually ameliorated fibrosis development by negatively regulating Th2-dependent responses [217]. This contradictory finding highlights other new findings that also suggest that M2 macrophages could be antifibrotic. A mechanistic study in a model of Schistosoma-induced liver fibrosis with specific deletion of the IL-4R*α* on myeloid cells preventing alternative activation of macrophages showed that M2 macrophages are not required for fibrosis development [218]. In addition, related studies with mice lacking arginase-1 in M2 macrophages showed that the arginase-1-expressing M2 macrophages were required for suppression and resolution of fibrosis [217]. This correlates well with findings that uptake of ECM components appears to be mediated by M2 macrophages. Uptake of these components is mediated by different mannose receptors [27] and by glycoprotein milk fat globule epidermal growth factor 8 (Mfge8) [219]. Mannose receptors of course are a known M2 marker, and for Mfge8 this is unclear. Both mannose receptor 2 and Mfge8 were shown to attenuate fibrosis in different models [219, 220].

To summarize, M2 macrophages are firmly associated with fibrosis development, but new evidence suggests they may actually contribute to resolution of fibrosis. Their presence during fibrosis may be explained as a failing attempt to clear the excess ECM. The conflicting roles described in the literature may be the result of difficulties separating the effects of M2 and M2-like macrophages simply because these two subsets are difficult to distinguish. M2-like macrophages may be a more likely candidate for the promotion of fibrosis as will be discussed below.

### 5.6. M2-Like Macrophages in Pulmonary Fibrosis

The specific role of M2-like macrophages has not been investigated in lung fibrosis yet, but M2-like macrophages may be important during the transition from inflammation towards tissue healing. The signature marker of M2-like macrophages is IL-10, which is the canonical anti-inflammatory cytokine with profibrotic actions. Elevated levels of IL-10 and enhanced production of IL-10 by alveolar macrophages have been reported in several fibrotic diseases, including IPF [221–223] and in systemic sclerosis patients with interstitial lung disease [213]. Its anti-inflammatory actions in lung are illustrated by a study from Armstrong et al., showing that IL-10 inhibited TNF*α* production by alveolar macrophages after LPS stimulation [224]. In addition, several studies in mice using the model of bleomycin-induced fibrosis suggest that IL-10 attenuates bleomycin-induced inflammation and can thereby attenuate fibrosis development [182, 225, 226]. However, overexpression of IL-10 in lungs of mice was found to be profibrotic [227]. Sun et al. found that inducible IL-10 overexpression in Clara cells induced fibrosis by fibrocyte recruitment and activation of macrophages towards an M2 phenotype. The increased levels of IL-10 found in lungs of IPF patients may therefore contribute to the fibrotic process. The production of the profibrotic, anti-inflammatory cytokine TGF*β* would also fit with this role of dampening inflammation and promoting tissue repair by this subset of macrophages. Whether TGF*β* production is restricted to the IL-10-producing M2-like macrophage subtype remains to be investigated.

In summary, M2-like macrophages are likely candidates for promotion of fibrosis. They may be recruited or induced by damage to the epithelium to dampen inflammation and start repair. In the event of ongoing damage they are continually induced or recruited and may contribute to fibrosis by overexpression of IL-10. Since corticosteroids are also capable of inducing M2-like macrophages, this would explain why these drugs are not effective against fibrosis and may even be disadvantageous. This is illustrated by our finding that when corticosteroids are specifically delivered to liver macrophages in a model of liver fibrosis, fibrosis actually becomes worse [228].

**Figure 4:**
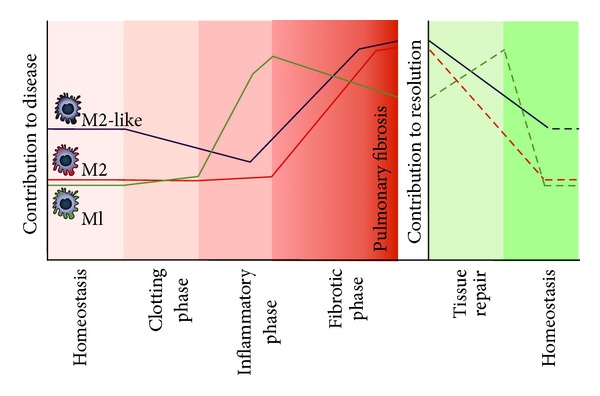
Schematic representation of the presence of M1, M2, and M2-like macrophages in lung tissue during homeostatic conditions and after injury to the lung. Normally after lung injury a process of tissue repair is initiated with four distinct phases leading to homeostatic conditions again. In lung fibrosis this normal tissue repair response is dysregulated leading to deposition of excess extracellular matrix and little resolution of scar tissue.

Overall, current data on the role of macrophages in the development of pulmonary fibrosis show that macrophages are important cells in the pathogenesis of this disease (see also Figure 4). M1 macrophages are important in the inflammatory phase and may also be important for resolution of the disease, although this hypothesis needs testing. M2 and M2-like macrophages are highly associated with fibrogenesis. However, new data suggest that M2 macrophages may actually protect against development of fibrosis while M2-like macrophages contribute to fibrosis. Therefore, key to understanding how these two phenotypes contribute to pulmonary fibrosis are studies differentiating between M2 and M2-like macrophages.

## 6. Conclusion

The literature on lung macrophages summarized in this paper clearly shows that macrophages are important in maintaining tissue homeostasis in the lung. Through their ability to change phenotypes they are able to regulate responses to homeostatic threats without impairing the functionality of the organ. The available literature also shows that when phenotype switching becomes dysfunctional or when some aspects of a particular phenotype become dysfunctional, pathologies develop. However, data on the distribution of macrophage subsets in healthy lung tissue and during disease is sorely lacking for humans as well as experimental models of respiratory diseases.

In general, asthma, COPD, and pulmonary fibrosis are diseases characterized by changes in macrophage subsets in the lung (M1, M2, and M2-like). It seems likely that changes in the interactions between the different subsets, that is, the balance, and changes in their function are a cause for disease, rather than the presence of one particular subset. The next challenge will be to specifically improve a particular function of a subset *in vivo* or specifically change a phenotype as a novel therapeutic approach for obstructive and restrictive respiratory diseases.
